# Evaluation of AI-Driven LabTest Checker for Diagnostic Accuracy and Safety: Prospective Cohort Study

**DOI:** 10.2196/57162

**Published:** 2024-08-14

**Authors:** Dawid Szumilas, Anna Ochmann, Katarzyna Zięba, Bartłomiej Bartoszewicz, Anna Kubrak, Sebastian Makuch, Siddarth Agrawal, Grzegorz Mazur, Jerzy Chudek

**Affiliations:** 1 Department of Internal Medicine and Oncological Chemotherapy, Medical University of Silesia, Reymonta St. 8, Katowice, 40-027, Poland, +48 32 2591 202; 2 Labplus R&D, Wroclaw, Poland; 3 Department of Clinical and Experimental Pathology, Wroclaw Medical University, Wroclaw, Poland; 4 Department and Clinic of Internal Medicine, Occupational Diseases, Hypertension and Clinical Oncology, Wroclaw Medical University, Wroclaw, Poland

**Keywords:** LabTest Checker, CDSS, symptom checker, laboratory testing, AI, assessment, accuracy, artificial intelligence, health care, medical fields, clinical decision support systems, application, applications, diagnoses, patients, patient, medical history, tool, tools

## Abstract

**Background:**

In recent years, the implementation of artificial intelligence (AI) in health care is progressively transforming medical fields, with the use of clinical decision support systems (CDSSs) as a notable application. Laboratory tests are vital for accurate diagnoses, but their increasing reliance presents challenges. The need for effective strategies for managing laboratory test interpretation is evident from the millions of monthly searches on test results’ significance. As the potential role of CDSSs in laboratory diagnostics gains significance, however, more research is needed to explore this area.

**Objective:**

The primary objective of our study was to assess the accuracy and safety of LabTest Checker (LTC), a CDSS designed to support medical diagnoses by analyzing both laboratory test results and patients’ medical histories.

**Methods:**

This cohort study embraced a prospective data collection approach. A total of 101 patients aged ≥18 years, in stable condition, and requiring comprehensive diagnosis were enrolled. A panel of blood laboratory tests was conducted for each participant. Participants used LTC for test result interpretation. The accuracy and safety of the tool were assessed by comparing AI-generated suggestions to experienced doctor (consultant) recommendations, which are considered the gold standard.

**Results:**

The system achieved a 74.3% accuracy and 100% sensitivity for emergency safety and 92.3% sensitivity for urgent cases. It potentially reduced unnecessary medical visits by 41.6% (42/101) and achieved an 82.9% accuracy in identifying underlying pathologies.

**Conclusions:**

This study underscores the transformative potential of AI-based CDSSs in laboratory diagnostics, contributing to enhanced patient care, efficient health care systems, and improved medical outcomes. LTC’s performance evaluation highlights the advancements in AI’s role in laboratory medicine.

## Introduction

In recent times, the implementation of artificial intelligence (AI) within diverse medical domains has garnered significant attention and practical application [1]. AI-driven technology has sparked a transformative wave in health care, introducing inventive solutions to enhance patient care, diagnosis, and decision-making processes [2]. A notable instance of AI’s application is evident in the emergence of clinical decision support systems (CDSSs), direct tools designed to streamline health care decision-making [3].

Laboratory tests are essential in modern health care, providing valuable insight into a patient’s health status and improving the accuracy of diagnosing medical conditions. The interpretation of laboratory test results is a complex process requiring medical expertise and knowledge. However, the mounting reliance on laboratory testing poses a formidable challenge for health care systems, particularly in regions where tests are often administered without direct medical oversight, as seen in Poland [4].

The significance of this challenge is highlighted by the substantial volume of inquiries related to laboratory test result interpretation, which include identifying potential causes or implications of certain findings and seeking guidance on the next steps or actions based on the test results. Data indicate that, in Poland alone, there are approximately 7 million monthly searches concerning the significance of laboratory test results. On a larger scale, within the European Union, this number escalates to around 82 million monthly searches based on data from SENUTO and Google AdWords [5]. These cases emphasize the need for effective strategies to manage laboratory test interpretation in modern health care settings.

Given the widespread use of laboratory diagnostics, which includes a wide range of tests that analyze blood, urine, tissues, and other bodily fluids to diagnose and monitor diseases, assess organ function, and guide treatment decisions, there is a growing interest in exploring the potential of CDSSs within this realm. The inherent complexities tied to test result interpretation underscore this interest. While the efficacy and safety of CDSSs have been demonstrated in various medical contexts, such as symptom assessment tools [6,7], the integration of CDSSs into laboratory diagnostics remains underexplored.

Several studies have assessed the effectiveness and safety of AI-driven symptom checkers, tools designed to aid patients in self-diagnosing symptoms and making informed health care choices [8-10]. These tools use algorithms and databases to generate potential diagnoses based on user inputs.

A notable study conducted by Semigran et al [11] scrutinized the diagnostic precision of 23 distinct symptom checkers, comparing their outcomes against physician diagnoses. The investigation disclosed that symptom checkers achieved accurate diagnoses in 34% of instances, while physicians achieved 58% accuracy. Despite relatively lower accuracy, the study underscored the potential of symptom checkers in offering reasonable differential diagnoses and supporting patient decision-making.

A more recent study by Hennemann et al [12] evaluated the performance of an app-based symptom checker within the realm of mental disorders. Results revealed that the studied symptom checker demonstrated moderate-to-good accuracy in suggesting conditions for mental disorders concerning formal diagnosis, albeit with variations across disorder categories and interrater reliability. The symptom checker’s primary condition suggestion corresponded with interview-based diagnoses in 51% (25/49) of cases, with at least 1 of the initial 5 condition suggestions aligning in 69% (34/49) of cases across the patient cohort. Accuracy fluctuated across disorder categories, ranging from 82% precision for somatoform and related disorders, 65% for affective disorders, to 53% for anxiety disorders. The study concluded that symptom checkers hold promise as supplementary screening tools in the diagnostic process. Still, their diagnostic efficacy requires assessment in more extensive samples and comparison with alternative diagnostic methods.

This paper addresses the status of AI-based technologies in health care, specifically focusing on implementing CDSSs in direct-to-patient tools. After emphasizing the importance of laboratory diagnostics in contemporary health care and the challenges tied to test result interpretation, we examine the existing but limited literature concerning CDSSs’ role in laboratory diagnostics, underscoring the need for further research and advancement in this domain. The objective of this study is to evaluate the performance of a novel CDSS named LabTest Checker (LTC) in a cohort of adult patients requiring laboratory testing. The main question it aims to answer pertains to the accuracy and safety of LTC.

## Methods

### Description of LTC Technology

LTC is an intricate medical software designed to provide assistance in the preliminary medical diagnosis process through the analysis of laboratory test results and comprehensive medical history. By leveraging advanced white-box machine learning algorithms and data analytics, LTC empowers patients and health care practitioners to derive insightful conclusions and make informed decisions. The AI models were trained on a comprehensive dataset encompassing clinical data from electronic health records, public repositories, documented case studies, and expert medical knowledge. LTC seamlessly integrates with existing electronic health record systems, automatically importing patients’ latest laboratory results. This triggers a dynamic medical questionnaire presented on a user-friendly tablet interface, typically completed within 90‐120 seconds, which delves into the patient’s medical history, symptoms, and pertinent risk factors. Through this methodical scrutiny and correlation of pivotal data, LTC effectively evaluates an individual’s health status and detects potential medical issues, empowering patients and health care providers to establish more accurate diagnoses and improve patient care and outcomes.

### Study Setting and Population

The study was conducted at the Emergency Department (ED) of Andrzej Mielecki Public Clinical Hospital in Katowice, Poland, with 8 specialized departments and a total bed capacity of 351. This cohort study embraced a prospective data collection approach. A total of 101 self-referred patients aged ≥18 years, in stable condition, but requiring comprehensive diagnosis were enrolled between December 22, 2022, and March 31, 2023. Comprehensive diagnosis refers to cases where diagnosis based solely on subjective evaluation and physical examination is unattainable, necessitating in-depth assessment through laboratory tests. Inclusion criteria encompassed (1) age ≥18 years and (2) requirement of in-depth laboratory test investigation. The only exclusion criteria was pregnancy. Trained research staff identified and invited eligible patients to participate following an initial medical evaluation to assess eligibility based on the predefined inclusion and exclusion criteria (further details are available on ClinicalTrials.gov [13]). The study achieved a high response rate of 84.9% (101/119), indicating strong participant willingness.

### Study Design

This prospective cohort study involved 101 patients, all requiring comprehensive diagnosis beyond subjective evaluation and physical examination. A panel of blood laboratory tests exceeding the routine diagnostic work-up at the ED, including a lipid profile, erythrocyte sedimentation rate, high-sensitivity C-reactive protein, creatinine, urea, iron, liver enzymes (alanine transaminase, aspartate transferase, and gamma-glutamyl transferase), sodium, potassium, glucose, uric acid, thyroid-stimulating hormone, and complete blood count, was conducted for each participant. LTC was used to interpret these results, and its performance was compared to that of an internal medicine specialist (JC), who reviewed the urgency categorizations assigned by ED physicians and the AI-generated suggestions without prior knowledge of the LTC results. It is important to clarify that JC did not directly assess patients in the ED. Instead, attending physicians in the ED assigned the initial urgency category for each patient based on their clinical judgment. JC then reviewed these urgency classifications assigned by the ED physicians, alongside the assessments generated by the model under study. This 2-pronged approach aimed to ensure the accuracy of the urgency categorizations and provide an additional layer of validation.

Patients presenting at the ED underwent laboratory tests and provided health-related information under a doctor’s supervision. This encompassed biometric details, medical history, medications, substances used, family history, symptoms, and prior test results. Based on these data and test outcomes, AI algorithms suggested underlying pathology and diagnostic-therapeutic guidance.

Accuracy and safety were assessed by comparing AI-generated suggestions to experienced doctor (consultant) recommendations, which are considered the gold standard. The consultant, blinded to the LTC results, categorized the urgency of physician interaction for each test (emergency, urgency, routine, and self-care; Table 1). Following this assessment, the LTC results were disclosed to the consultant to evaluate if adhering to LTC recommendations could avoid needless medical visits and whether LTC correctly identified the underlying causes of any abnormal results.

**Table 1.** Diagnostic and therapeutic recommendations generated by LabTest Checker (LTC) and specialist recommendations were categorized to assess LTC’s precision. Sensitivity for the emergency category was computed as the ratio of LTC’s correct emergency identifications to the physician’s emergency identifications: *A / (A + B + C + D)*. Similarly, sensitivity for the urgency category was calculated as *F / (E + F + G + H)*. Triage accuracy was calculated as *(A + F + L + R) / total number of patients in the study*. Triage safety as calculated as *(A + E + F + J + K + L + N + O + P + R) / total number of patients in the study*.

Urgency category of contact with doctor, assigned by the consultant	Urgency category of contact with doctor, assigned by LTC			
	Emergency	Urgency	Routine	Self-care
Emergency	A	B	C	D
Urgency	E	F	G	H
Routine	J	K	L	M
Self-care	N	O	P	R

Owing to the technology’s design, certain variables were excluded from determining pathology identification accuracy: (1) interpretations labeled as urgent or requiring immediate contact with a doctor were omitted to ensure patient safety and prioritize triage in emergencies, and (2) interpretations categorized as “end of diagnostic - no need for doctor contact” were omitted when results were valid or deviations were insignificant and did not signify pathology.

### Ethical Considerations

The study protocol was registered on ClinicalTrials.gov (NCT05813938), and ethical approval was granted by the Bioethics Committee of the Medical University of Silesia (approval code: PCN/CBN/0052/KB1/115/I/22; approval date: November 8, 2022). All patients provided written informed consent before undergoing screening for study eligibility. To ensure privacy and confidentiality, all data collected during the study were anonymized and deidentified before analysis. Participants received no compensation for their involvement in the study. The study involved noninvasive procedures, and the primary intervention was the use of LTC to interpret laboratory test results. Participants were informed that they could seek clarification or assistance from medical professionals while using LTC.

### Statistics

A power analysis was performed to determine the statistical power of this study, considering a total sample size of 101 participants in a single group of patients, which was predetermined in the study design. The power analysis was conducted using the G*Power software (version 3.1.9.7; Heinrich-Heine-Universität Düsseldorf). The power analysis was based on a 1-tailed test with an α level of .05. The effect size was calculated at 0.36. Using these parameters and the total sample size of 101, the power analysis indicated that the study would have moderate statistical power to detect a significant effect size within a single group of patients. The estimated power achieved with the given sample size was 0.82, indicating that the study had a reasonable likelihood of detecting meaningful differences within the group.

Outcome measures were prespecified and calculated with 95% CIs. The Wilson score method was used to produce CIs for sensitivity to emergency, sensitivity to urgency, accuracy of triage, safety of triage, and reduction of unnecessary visits. Calculations were performed using the statistical software package *Statistica* (version 13.0 PL; TIBCO Software Inc). Analytic data are presented as point estimates and 95% CIs, with a *P* value <.05 being considered significant.

## Results

In the context of this study, the triage accuracy in the 101-patient cohort was 74.3%, with a safety sensitivity of 100% for identifying emergency cases and a sensitivity of 92.3% for detecting urgent cases. The implementation of the system led to a noteworthy 41.6% (42/101) reduction in unnecessary medical visits, and its accuracy in identifying the underlying pathology was 82.9%.

The system classified patients based on urgency: 9 patients required immediate contact; 41 needed urgent contact; 50 warranted routine contact; and 1 did not necessitate doctor contact, falling into the self-care category. Analysis by the consultant revealed disparities in urgency category assignments for 26 patients. Notably, the technology overestimated urgency for 25 patients, including cases where the consultant recommended urgent contact, but the technology indicated immediate or scheduled contact. However, the technology inaccurately assessed the urgency for 1 patient, failing to align with the specialist’s urgent contact suggestion, instead proposing scheduled contact. These findings collectively underscore the triage system’s effective urgency categorization while also pinpointing areas for enhancement to improve precision, diminish disparities, and prevent false negatives. These findings are detailed in Table 2.

**Table 2.** Classification outcomes of diagnostic-therapeutic recommendations proposed by LabTest Checker (LTC) and those provided by the consultant.

Urgency category of contact with doctor, assigned by the consultant	Urgency category of contact with doctor, assigned by LTC			
	Emergency, n	Urgency, n	Routine, n	Self-care, n
Emergency	7	0	0	0
Urgency	1	24	1	0
Routine	1	15	43	0
Self-care	0	2	6	1

## Discussion

### Principal Findings

The promising results obtained from the evaluation of LTC show the potential of AI-driven tools in assisting patients and medical professionals in navigating the complexities of laboratory test result interpretation. An accuracy rate of 74.3% demonstrates LTC’s capability to furnish dependable medical recommendations grounded in blood test results, a development that holds promise for enhancing operational efficiency in the medical domain. Particularly noteworthy is LTC’s impressive safety sensitivity of 100% for identifying emergency cases and a high sensitivity of 92.3% for detecting urgent cases. These results imply the system’s adeptness in identifying critical scenarios, aligning with its intended role of providing secure and precise medical counsel.

### Comparison to Prior Work and Broader Implications

The clinical implications of our findings extend beyond acute care settings. LTC has the potential to revolutionize health care delivery across various domains, including outpatient clinics, preventive care, and direct-to-consumer health management.

In outpatient settings, LTC could streamline triage processes by providing rapid, accurate assessments of laboratory results, allowing health care providers to prioritize patients more effectively and potentially reducing the burden on overstretched health care systems. Additionally, integrating LTC into preventive care programs could empower individuals to monitor their health proactively, fostering early detection and intervention for potential health issues.

The potential impact of LTC on the direct-to-consumer health landscape is equally promising. By providing individuals with accessible and easily understandable interpretations of their laboratory results, LTC could facilitate informed decision-making, leading to earlier detection and more effective management of health conditions. This shift toward proactive health care could encourage individuals to take greater ownership of their well-being.

While the potential of AI-driven CDSSs such as LTC is evident, it is important to acknowledge the nascent nature of this field. One such study by Gräf et al [14] compared physician and AI-based symptom checker diagnostic accuracy, where the AI achieved a diagnostic accuracy of 70%. Furthermore, a systematic review of 10 studies revealed consistently low diagnostic accuracy (range 19%‐37.9%), while triage accuracy (range 48.8%‐90.1%) was relatively higher but displayed variability among different symptom checkers [9]. Our study attempted to adhere to established reporting guidelines for machine learning models [15], but more robust research is necessary to fully understand the capabilities and limitations of AI-powered CDSSs in diverse health care settings.

### Strengths and Limitations

While the study yielded promising results, several inherent limitations should be acknowledged when assessing the accuracy and safety of LTC. First, the sample size was relatively small, comprising only 101 participants. Although efforts were taken to ensure analytical strength, a larger and more diverse sample would enhance the generalizability of findings to the broader population. Furthermore, the study allowed participants to seek guidance from medical professionals when faced with uncertainties while filling out the questionnaire, which might not mirror real-world use where such guidance might not be readily accessible. While this provision was aimed at optimizing data quality, it could have potentially introduced an artificial element, warranting caution when considering the practical implications of the technology’s recommendations.

Unexpected results during the experiments included occasional discrepancies between LTC and consultant assessments, indicating potential areas for model improvement. These discrepancies could stem from complex interactions between predictor variables that were not fully captured by the model, suggesting the need for further refinement of the AI algorithms.

These limitations underscore the necessity for future research involving more representative samples and real-world use scenarios to validate the robustness and effectiveness of emerging CDSS technologies. By exploring the intersection of AI and laboratory diagnostics, we aim to lay the groundwork for future progress and foster a deeper comprehension of AI-based CDSSs’ potential in reshaping laboratory medicine.

### Conclusions

In conclusion, our study demonstrates the transformative potential of integrating AI into laboratory diagnostics through the LTC software. The high accuracy and safety sensitivity achieved underscore the ability of AI-driven CDSSs to identify medical conditions and provide tailored recommendations, enhancing health care decision-making.

As AI continues to evolve within health care, this study validates the promise of AI in medical diagnostics and highlights the need for continued research to refine and expand such tools. By fostering collaboration between AI experts and laboratory medicine specialists, we can unlock the full potential of AI-powered CDSSs, paving the way for a more efficient, personalized, and patient-centered approach to health care.

The results presented here offer a compelling glimpse into a future where AI-driven tools play a pivotal role in optimizing patient care and revolutionizing laboratory diagnostics.

## Abbreviations

AI: artificial intelligence
CDSS: clinical decision support system
ED: emergency department
LTC: LabTest Checker

## References

[1] Davenport T, Kalakota R (2019). The potential for artificial intelligence in healthcare. Future Healthc J.

[2] Johnson KB, Wei WQ, Weeraratne D (2021). Precision medicine, AI, and the future of personalized health care. Clin Transl Sci.

[3] Castaneda C, Nalley K, Mannion C (2015). Clinical decision support systems for improving diagnostic accuracy and achieving precision medicine. J Clin Bioinforma.

[4] Nagel A (2019). Polacy leczą się sami. 90 proc. zażywa leki bez recepty [Article in Polish]. WP abcZdrowie.

[5] Pokańko K (2022). Polskie AI ma w kilka sekund zweryfikować wyniki badań. wszystko online i bez wychodzenia z domu [Article in Polish]. Spider’s Web.

[6] Fraser H, Coiera E, Wong D (2018). Safety of patient-facing digital symptom checkers. Lancet.

[7] Sutton RT, Pincock D, Baumgart DC, Sadowski DC, Fedorak RN, Kroeker KI (2020). An overview of clinical decision support systems: benefits, risks, and strategies for success. NPJ Digit Med.

[8] Chambers D, Cantrell AJ, Johnson M (2019). Digital and online symptom checkers and health assessment/triage services for urgent health problems: systematic review. BMJ Open.

[9] Wallace W, Chan C, Chidambaram S (2022). The diagnostic and triage accuracy of digital and online symptom checker tools: a systematic review. NPJ Digit Med.

[10] Nateqi J, Lin S, Krobath H (2019). From symptom to diagnosis—symptom checkers re-evaluated: are symptom checkers finally sufficient and accurate to use? an update from the ENT perspective [Article in German]. HNO.

[11] Semigran HL, Linder JA, Gidengil C, Mehrotra A (2015). Evaluation of symptom checkers for self diagnosis and triage: audit study. BMJ.

[12] Hennemann S, Kuhn S, Witthöft M, Jungmann SM (2022). Diagnostic performance of an app-based symptom checker in mental disorders: comparative study in psychotherapy outpatients. JMIR Ment Health.

[13] Assessment of accuracy and safety of LabTest Checker (LTC-AI). ClinicalTrials.gov.

[14] Gräf M, Knitza J, Leipe J (2022). Comparison of physician and artificial intelligence-based symptom checker diagnostic accuracy. Rheumatol Int.

[15] Luo W, Phung D, Tran T (2016). Guidelines for developing and reporting machine learning predictive models in biomedical research: a multidisciplinary view. J Med Internet Res.

